# Machine learning-based prediction model and visual interpretation for prostate cancer

**DOI:** 10.1186/s12894-023-01316-4

**Published:** 2023-10-14

**Authors:** Gang Chen, Xuchao Dai, Mengqi Zhang, Zhujun Tian, Xueke Jin, Kun Mei, Hong Huang, Zhigang Wu

**Affiliations:** 1 School of Public Health and Management, Wenzhou Medical University, Wenzhou, 325035 China; 2 School of Environmental Science and Engineering, Suzhou University of Science and Technology, Suzhou, 215009 China; 3 Center for Health Assessment, Wenzhou Medical University, Wenzhou, 325035 China; 4 Zhejiang Provincial Key Laboratory of Watershed Sciences and Health, Wenzhou, 325035 China; 5 Department of Urology, The First Affiliated Hospital of Wenzhou Medical University, Wenzhou, 325035 China; 6 Reproductive Health Research Center, Health Assessment Center of Wenzhou Medical University, Wenzhou, 325000 China

**Keywords:** Prostate cancer, Machine learning, Shapley values, Biochemical parameters, Risk threshold

## Abstract

**Background:**

Most prostate cancers(PCa) rely on serum prostate-specific antigen (PSA) testing for biopsy confirmation, but the accuracy needs to be further improved. We need to continue to develop PCa prediction model with high clinical application value.

**Methods:**

Benign prostatic hyperplasia (BPH) and prostate cancer data were obtained from the Chinese National Clinical Medical Science Data Center for retrospective analysis. The model was constructed using the XGBoost algorithm, and patients’ age, body mass index (BMI), PSA-related parameters and serum biochemical parameters were used as model variables. Using decision analysis curve (DCA) to evaluate the clinical utility of the models. The shapley additive explanation (SHAP) framework was used to analyze the importance ranking and risk threshold of the variables.

**Results:**

A total of 1915 patients were included in this study, including 823 (43.0%) were BPH patients and 1092 (57.0%) were PCa patients. The XGBoost model provided better performance (AUC 0.82) compared with f/tPSA (AUC 0.75),tPSA (AUC 0.68) and fPSA (AUC 0.61), respectively. Based on SHAP values, f/tPSA was the most important variable, and the top five most important biochemical parameter variables were inorganic phosphorus (P), potassium (K), creatine kinase MB isoenzyme (CKMB), low-density lipoprotein cholesterol (LDL-C), and creatinine (Cre). PCa risk thresholds for these risk markers were f/tPSA (0.13), P (1.29 mmol/L), K (4.29 mmol/L), CKMB ( 11.6U/L), LDL-C (3.05mmol/L) and Cre (74.5-99.1umol/L).

**Conclusion:**

The present model has advantages of wide-spread availability and high net benefit, especially for underdeveloped countries and regions. Furthermore, these risk thresholds can assist in the diagnosis and screening of prostate cancer in clinical practice.

## Introduction

Prostate Cancer (PCa) is a common tumor of the urinary system and one of the major malignant tumors threatening men’s health in the world [1]. For instance, the incidence of prostate cancer in China is showing a continuous increase and therefore is gradually affecting men’s health [2]. In order to provide prostate cancer patients with a better prognosis and further improve their quality of life, early screening and accurate diagnosis of prostate cancer have become the focus of current study.

Prostate-specific antigen (PSA) is the most common tumor marker used for prostate cancer screening [3]. After PSA screening, a positive biopsy of the prostate is required to confirm the diagnosis of prostate cancer. However, PSA may be elevated in patients with benign prostatic hyperplasia (BPH), prostatitis, or other non-prostate cancer. The use of PSA as the sole tool for prostate biopsy decisions has led to a high number of overdiagnosis of inert prostate cancer. To improve the accuracy of screening systems, various methods have been introduced in predicting prostate cancer, such as measurement of PSA derivatives, PSA kinetics and mpMRI [4, 5]. However, these new techniques have limited performance in improving the diagnosis of prostate cancer. Therefore, the search for new markers for prostate cancer risk assessment continues.

Machine learning (ML) techniques have been widely used in clinical medicine, especially in building predictive models, and various machine learning techniques have been used to enhance prostate cancer prediction, showing stronger performance than traditional predictive models [6, 7]. However, they are often criticized due to their lack of interpretability and “black box” nature. The lack of intuitive model interpretation is considered a major limitation to the practical adoption of ML models by clinicians. In order to improve the interpretability of complex ML models, the SHapley Additive exPlanations (SHAP) framework has been proposed, which represents a unified approach to interpreting the predictions of complex ML models [8]. SHAP value is a way to describe the “weight” or “importance” that a model applies to a particular feature when predicting a particular data point, with a positive or negative value indicating the direction of influence.

In this study, we constructed a XGBoost model using machine learning method to distinguish PCa and BPH patients. In addition to patient demographics and traditional PSA-related indicators, widely available pre-biopsy serum biochemical information was used as input for model construction. The SHAP framework was used to visually interpret the relationship between each variable and prostate cancer and to obtain the corresponding risk thresholds.

## Materials and methods

### Materials

The Chinese National Clinical Medicine Science Data Center (https://www.ncmi.cn), one of the data centers of the National Population Health Science Data Sharing Platform, is jointly undertaken by Peking Union Medical College Hospital and Chinese PLA General Hospital (301 Hospital). The data for this study were obtained from the Prostate Tumor Warning Dataset of the Chinese PLA General Hospital (301 Hospital). After excluding cases with missing data points, a total of 1,915 Chinese male cases were included in this study, and all patients underwent prostate biopsy, including 23 predictor variables and 1 diagnostic outcome. In this study, the 23 predictor variables recorded for each patient included age, body mass index (BMI), serum albumin (ALB), alkaline phosphatase (ALP), creatine kinase MB isoenzyme (CKMB), free PSA (fPSA), total PSA (tPSA), free-to-total PSA ratio (f/tPSA), sodium (Na), calcium (Ca), chloride (Cl), inorganic phosphorus (P), free calcium (fCa), lactate dehydrogenase (LDH), creatine kinase (CK), creatinine (Cre), uric acid (UA), triglycerides (TG), high-density lipoprotein cholesterol (HDL-C), low-density lipoprotein cholesterol (LDL-C), apolipoprotein A1 (Apo-A1), apolipoprotein B (Apo-B), and potassium (K).

### Methods

The XGBoost [9] used in this study is a powerful model, a variant of the Gradient Boost Machine (GBM). The patient data were randomly divided into training and test sets in the ratio of 7:3. The training set was used to build models and the test set was used for model validation and evaluation. Accuracy, sensitivity, specificity and area under the receiver operating characteristic curve (AUC) were calculated to evaluate the model performance. The 95% confidence interval (CI) and comparisons of AUCs were determined using the method of DeLong et al. [10]. Decision curve analyses (DCA) [11] were used to compare the net benefit of different models. The SHAP framework was constructed for the established XGBoost model, and the SHAP values were used to rank the importance of the predictor variables. Based on the SHAP values, the relationship between the variables and the risk of PCa were analyzed, and if the SHAP value > 0, it indicated that the variable elevated the predictive value, i.e., had a facilitative effect on the outcome, which in this study indicated an increased risk of PCa. Descriptive analyses and DCA were done in SPSS (version 25.0, IBM, USA) and R (version 4.0.4). Machine learning were performed using open-source libraries (Scikit-learn and SHAP) available in Python 3.7.

## Results

In this dataset, patients with incomplete data points were excluded and we identified 1915 patients for analysis. Of the total patient cohort, 823 patients (43.0%) had BPH and 1092 patients (57.0%) had PCa. Once divided into training and testing, this resulted in 1340 data points in the training set and 575 data points for the final test. The baseline characteristics of the patients were listed in Table 1.

**Table 1:** Baseline characteristics of the benign prostatic hyperplasia(BPH) and prostate cancer (PCa) patients

Variable	All patients (n = 1915)	BPH (n = 823)	PCa (n = 1092)
Age (year)	67(62–73)	69(62–74)	67(61–72)
BMI (Kg/m^2^)	24.74(22.72–26.67)	24.57(22.39–26.56)	24.82(23.03–26.73)
ALB (g/L)	41.2(39.10–43.30)	40.6(38.60–42.90)	41.5(39.60–43.50)
ALP (U/L)	63.3(53.9–75.1)	63.2 (53.9–74.5)	63.5(53.9–75.5)
CKMB (U/L)	13.6(10.7–16.5)	13.1(1.1–16.1)	13.9(11.20-16.78)
fPSA (ng/ml)	0.71(0.26–1.60)	0.82(0.38–1.68)	0.64(0.16–1.54)
tPSA (ng/ml)	5.24(1.68–12.40)	4.48(2.00-9.81)	6.16(1.34–14.90)
f/tPSA	0.15(0.09–0.24)	0.19(0.13–0.25)	0.11(0.07–0.21)
Na (mmol/L)	142.7(141.2-144.1)	142.6(141.1–144.0)	142.8(141.3-144.1)
Ca (mmol/L)	2.23(2.17–2.30)	2.22(2.16–2.28)	2.25(2.19–2.32)
Cl (mmol/L)	104.4(102.4-106.4)	104.6(102.4-106.7)	104.4(102.3-106.2)
P (mmol/L)	1.12(1.01–1.23)	1.09(0.98–1.19)	1.15(1.04–1.28)
fCa (mmol/L)	1.15(1.11–1.18)	1.14(1.10–1.17)	1.15(1.12–1.18)
LDH (U/L)	150.1(134.7-168.6)	150.2(135.2-166.8)	149.9(133.83–169.6)
CK (U/L)	79.8(59.6-107.2)	77.3(57.1–105.0)	82.4(61.7-108.4)
Cre (umol/L)	79.8(71.6–89.4)	79.7(71.7–89.9)	79.9(71.53-89.0)
UA (µmol/L)	328.2(275.8-379.9)	327.6(277.4-383.3)	328.6(274.4-378.8)
TG (mmol/L)	1.14(0.84–1.59)	1.09(0.83–1.51)	1.17(0.85–1.66)
HDL-C (mmol/L)	1.14(0.97–1.36)	1.14(0.95–1.33)	1.15(0.98–1.37)
LDL-C (mmol/L)	2.71(2.25–3.27)	2.62(2.18–3.09)	2.81(2.30–3.40)
Apo-A1 (g/L)	1.25(1.10–1.42)	1.22(1.07–1.39)	1.27(1.12–1.44)
Apo-B (g/L)	0.87(0.74–1.03)	0.85(0.72–0.99)	0.90(0.75–1.06)
K (mmol/L)	4.02(3.81–4.23)	4.03(3.81–4.27)	4.01(3.80–4.21)

Data are presented as median (inter-quartile range)

*BPH* benign prostatic hyperplasia, *PCa* prostate cancer, *BMI* body mass index, *ALB* serum albumin, *ALP* alkaline phosphatase, *CKMB* creatine kinase MB Isoenzyme, *fPSA* free prostate-specific antigen, *tPSA* total prostate-specific antigen, *f/tPSA* free-to-total PSA ratio, *Na* sodium, *Ca* calcium, *Cl* chloride, *P* inorganic phosphorus, *fCa* free calcium, *LDH* lactate dehydrogenase, *CK* creatine kinase, *Cre* creatinine, *UA* uric acid, *TG* triglyceride, *HDL-C* high density lipoprotein cholesterol, *LDL-C* low density lipoprotein cholesterol, *Apo-A1* Apolipoprotein A1, *Apo-B* Apolipoprotein B, *K* potassium

**Fig. 1:**
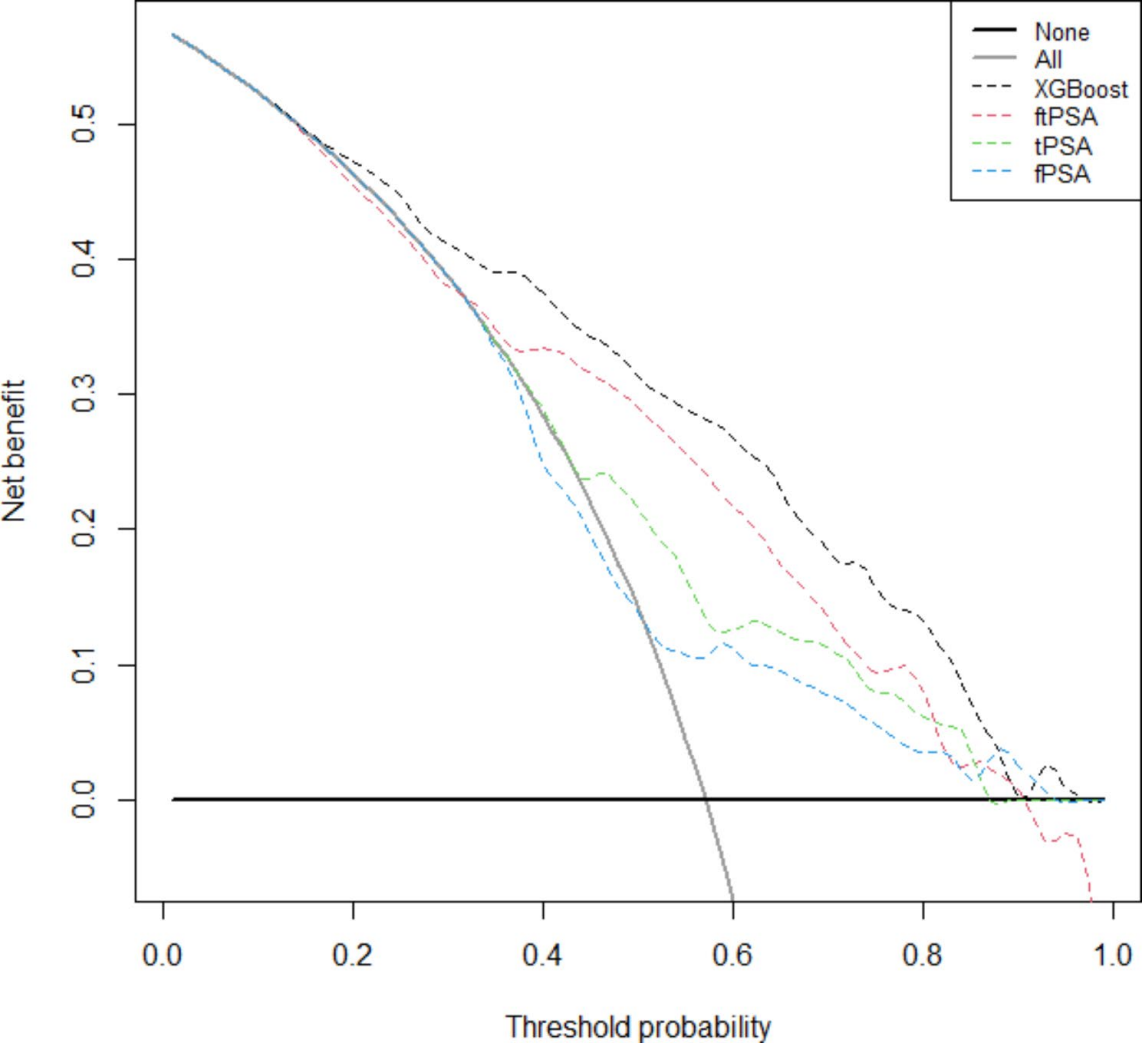
Decision curve analysis (DCA) of models in the validation cohort. *f/tPSA* free-to-total PSA ratio, *tPSA* total prostate-specific antigen, *fPSA* free prostate-specific antigen

To determine the validity of the model, we calculated the evaluation metrics using f/tPSA, tPSA, and fPSA as the sole determinants of classification, respectively (Table 2). When using AUC as a measure of predictive model performance, XGBoost had an AUC of 0.82, and the model outperformed the other models with a single variable. The AUC of XGBoost was significantly compared with that of f/tPSA, tPSA and fPSA models (each *P* < 0.001). We performed DCA using predictive risk in the validation cohort to evaluate the potential clinical benefits of each model. It was observed that the XGBoost model had higher net benefit than f/tPSA, tPSA and fPSA models across the threshold probabilities above 10% (Fig. 1).

**Table 2:** Performance comparison between univariate model and XGBoost model

Model	Accuracy(%)	Sensitivity(%)	Specificity(%)	AUC (95% CI)
XGBoost	74.09	79.57	71.12	0.82 (0.79–0.82)
f/tPSA	71.82	75.46	67.07	0.75 (0.72–0.76)*
tPSA	64.70	68.33	59.40	0.68 (0.65–0.70)*
fPSA	57.04	60.36	50.00	0.61 (0.58–0.63)*

* The AUC of XGBoost was significantly compared with that of f/tPSA, tPSA and fPSA models (each *P*<0.001)

*f/tPSA* free-to-total PSA ratio, *tPSA* total prostate-specific antigen, *fPSA* free prostate-specific antigen

**Fig. 2:**
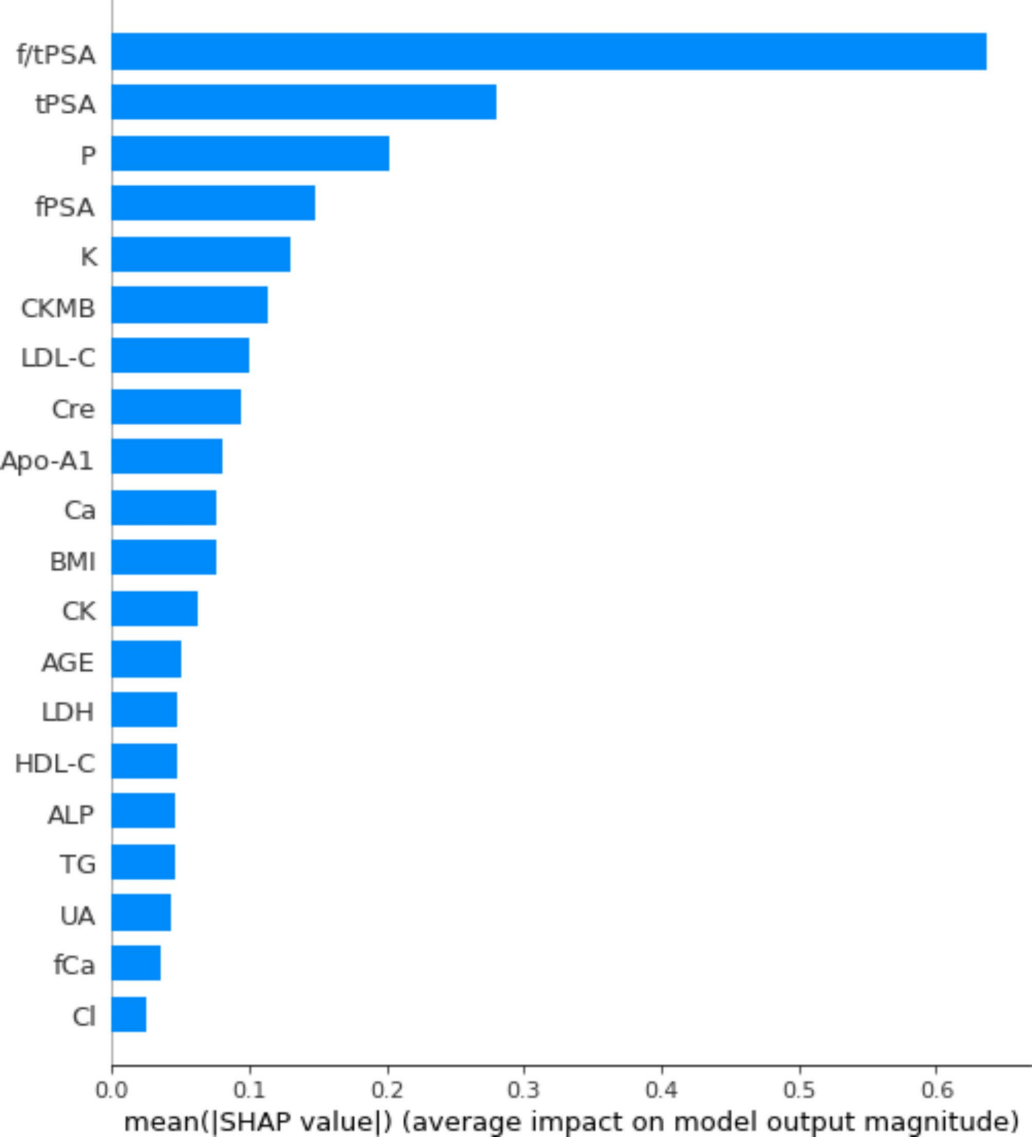
Ranking of input variables in the XGBoost model to predict prostate cancer (Based on SHAP values). *BMI* body mass index, *ALP* alkaline phosphatase, *CKMB* creatine kinase, *fPSA* free prostate-specific antigen, *tPSA* total prostate-specific antigen, *f/tPSA* free-to-total PSA ratio, *Ca* calcium, *Cl* chloride, *P* inorganic phosphorus, *CK* creatine kinase, *Cre* creatinine, *UA* uric acid, *TG* triglyceride, *HDL-C* high density lipoprotein cholesterol, *LDL-C* low density lipoprotein cholesterol, *Apo-A1* Apolipoprotein A1, *Apo-B* Apolipoprotein B, *K* potassium

**Fig. 3:**
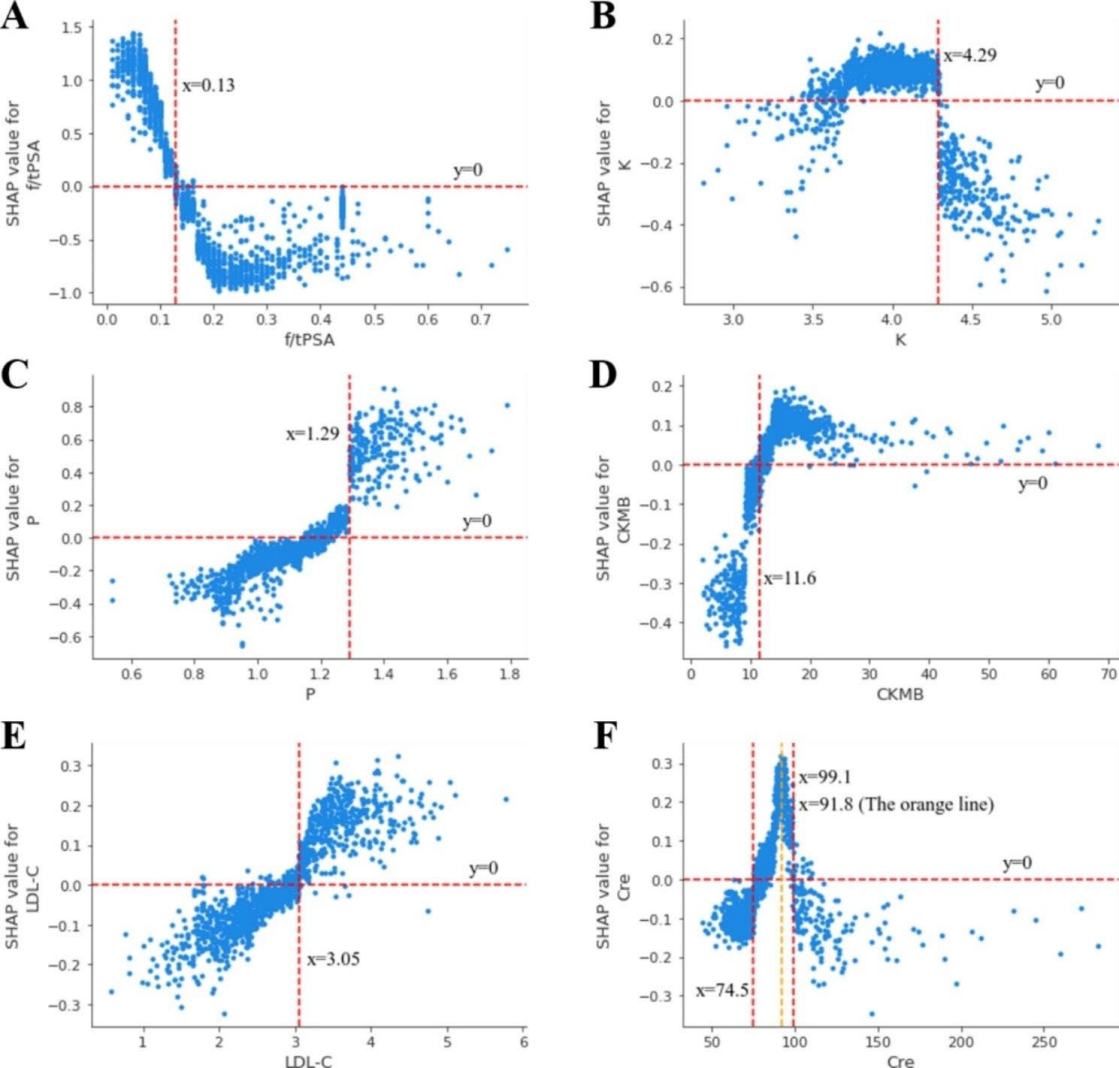
Shapley additive explanation (SHAP) dependence plots between prostate cancer risk and individual risk factors. (**A**) Dependence plot between free-to-total PSA ratio (f/t PSA) and SHAP value. (**B**) Dependence plot between potassium (K) and SHAP value. (**C**) Dependence plot between inorganic phosphorus (P) and SHAP value. (**D**) Dependence plot between creatine kinase MB Isoenzyme (CKMB) and SHAP value. (**E**) Dependence plot between low density lipoprotein cholesterol (LDL-C) and SHAP value. (**F**) Dependence plot between creatinine (Cre) and SHAP value

The importance of the variables in the XGBoost model for predicting PCa based on SHAP values were calculated (Fig. 2). It was clear that f/tPSA was the most important variable in the model, followed by tPSA. The top five biochemical parameter variables with the highest contribution to the model were P, K, CKMB, LDL-C, and Cre. We generated SHAP dependence plots for f/tPSA and the five most important biochemical parameter variables. The SHAP values gradually decrease with increasing f/tPSA and then remain constant with a threshold of 0.13 (Fig. 3A). When f/tPSA < 0.13, the SHAP values > 0, implying that low f/tPSA values had a positive effect on predicting PCa and patients were at greater risk of PCa. Similarly, patients with lower serum potassium concentration levels were more likely to be PCa patients, and the threshold for K was 4.29mmol/L (Fig. 3B). On the contrary, increasing values of P, CKMB and LDL-C showed a positive correlation with increasing SHAP values (Fig. 3C, D and E). The thresholds for P, CKMB and LDL-C were 1.29 mmol/L, 11.6 U/L and 3.05 mmol/L, respectively. The relationship between Cre and PCa risk showed a specific nonlinear relationship, with a cut-off point of 91.8umol/L, which had a risk threshold interval between 74.5-99.1umol/L (Fig. 3F).

## Discussion

Accurate prostate cancer risk assessment is essential to facilitate the diagnosis of prostate cancer while limiting the number of unnecessary prostate biopsies. A simple, easy-to-use, inexpensive and effective predictive model was attempted to be used to avoid a large number of unnecessary biopsies based on PSA alone in this study. Our model lies in the fact that the required clinical parameters are widely available and objective, without requiring extra costs and expertise in novel biomarkers and/or imaging. Such technique can be used to screen populations with the advantage of low cost and ease of scalability, especially for hospitals in less developed areas with poorer medical equipment, such as many rural hospitals in China. Thus, optimizing patients suitable for further diagnosis including mpMRI or prostate biopsy.

Machine learning model is a relatively new technique in its infancy in clinical practice, but have shown great promise for application in biomedical sciences [12]. The predictive model in our study adds to the limited body of evidence supporting machine learning techniques in urological practice. XGBoost, a powerful algorithm proposed in 2016, uses multiple strategies to prevent overfitting, exploits the second-order derivatives of the loss function and supports parallelization, and has fast data processing speed [9]. Liu et al. developed and validated several widely used machine learning algorithms to predict the risk of bone metastases in PCa patients and found that the XGBoost algorithm-based prediction model performed the best among all the prediction models [7]. In a recent study to find new biomarkers associated with metastasis and to predict breast cancer metastatic status, XGBoost model obtained a higher mean AUC than other classifiers [13]. When we used several major classical predictors as the sole determinants of classification, these models had lower accuracy, sensitivity, specificity and AUC than XGBoost model in this study. In a retrospective study, Perera et al. used a dense neural network (DNN) machine learning model incorporating age, PSA, free PSA, and free-to-total PSA ratio to improve the diagnosis of PCa, and showed an AUC of 0.72 for DNN compared to 0.65 (free-to-total PSA ratio) and 0.63 (PSA only) [14]. In comparison, our model (AUC of 0.82) is better than DNN model. However, this advantage should take into account the impact of different study cohorts. In decision curve analyses (Fig. 1), XGBoost model demonstrated net clinical benefit over f/tPSA, tPSA and fPSA across different threshold probabilities. In other words, using XGBoost model should be recommended for clinical use as it provides the highest clinical benefit. Our XGBoost model has higher specificity while having higher sensitivity compared to models that only incorporate f/t PSA. The purpose of the model is not to directly replace the results of puncture biopsy, but to recommend more real prostate cancer patients for puncture biopsy, to improve the positive rate of puncture biopsy, and to avoid excessive underdiagnosis and overdiagnosis.

The SHAP framework was used to explore the “black box” in machine learning. We identified a number of biochemical parameters with predictive value and indicated their thresholds that have the potential to become new indicators for screening prostate cancer. Unlike classical statistical models, where machine learning allows unbiased ranking of the relative importance of input variables, we used SHAP values to rank the degree of importance of the variables. The ranking in our model highlighted the importance of PSA-related parameters, in addition to the five biochemical parameters P, K, CKMB, LDL-C, and Cre as important predictors of prostate cancer. In the ranking graph of the importance of the variables, we can see that the degree of importance of f/tPSA is much higher than other variables, confirming that the free-to-total PSA ratio is still one of the most important predictors of prostate cancer in clinical practice, a result similar to that of a Japanese study [15].

A study conducted in a Chinese population in 2020 found that a free-to-total PSA ratio of 0.15 had better sensitivity and specificity in differential diagnosis of PCa and BPH. Similarly, we found that the model with a free-to-total PSA ratio threshold of 0.13 had the best performance. In a recent mendelian randomization and meta-analysis study, the researcher showed a potential causal relationship between circulating phosphorus and risk of prostate cancer, with high dietary phosphorus intake and elevated serum phosphorus concentration respectively, were associated with increased risk of prostate cancer [16]. This study supported our findings and further confirms the potential of serum inorganic phosphorus to predict PCa. The normal range of serum potassium (K) was 3.5–5.5 mmol/L [17], and the serum potassium of the patients in this study was basically within the normal range, even so, we found that the potential risk threshold for potassium was 4.29 mmol/L. Serum potassium concentrations are mainly associated with chronic kidney disease and heart failure [18]. No studies have been done to explore the relationship between potassium and prostate cancer, and our findings may provide a new direction for research. Earlier, A Gries et al. accidentally found that the amount of CKMB may be associated with prostate cancer [19]. Since then, based on the continuous development of proteomics, many scholars have suggested the inclusion of CKMB as a malignancy marker in clinical screening [20]. Our study provided new evidence that CKMB may be a risk factor for PCa with a threshold value of 11.6 U/L, similar to the results of Guo et al. [21]. The current discussion on the relationship between lipids and prostate cancer is still controversial, and we found that LDL-C is an important predictor of PCa. Similarly, a case-control study conducted by Magura et al. reported that high LDL-C may be a risk factor for PCa [22]. Several values have been used to define creatininemia, but thresholds are typically in the 1.5-2.0 mg/dL (132.6-176.8umol/L) range [23], and the vast majority of patients in this study had creatinine values in the normal range. A prospective study reported a strong association between higher serum creatinine in the normal range and higher risk of prostate cancer, and the correlation appeared to be dose-dependent [24]. We found a positive association between creatinine and PCa risk when creatinine values were less than 91.8umol/L, similar to the results of this prospective study. Interestingly, a negative correlation was observed when creatinine values were greater than 91.8umol/L. Previously, a nonlinear relationship between creatinine and PCa risk has not been reported, and we have proposed a risk threshold interval (74.5-99.1umol/L), but more studies are needed to validate it. Notably, the SHAP framework offers a promising method for interpreting predictions as well as visualizing nonlinear relationships in machine learning-based models in oncology.

Initial machine learning techniques in prostate cancer diagnosis were introduced by Snow et al. using PSA level, DRE and TRUS parameters [25]. Multiple subsequent iterations have been generated, mostly including DRE or TRUS parameters. However, these parameters are not considered first-line screening tests and may be considered subjective parameters with some degree of interobserver variability [26], thus limiting their clinical application. In fact, current guidelines from the United States Prevention Task Force (USPTF) and Urological Society of Australia and New Zealand (USANZ) do not support the routine use of DRE [27, 28]. These subjective parameters were excluded from our model, thus reducing the limitations of clinical use. In addition to parameter objectivity, potential generality and wide applicability are also the advantages of our model. Other novel biochemical markers for prostate cancer risk assessment such as the prostate health index (PHI) [29] and prostate cancer antigen 3 (PCA3) [30] may reduce unnecessary biopsies to some extent, but cost, convenience and accessibility are barriers to widespread clinical application. Using AUC as a predictor of performance, PHI was 0.70 [29] and PCA3 was 0.734 [30], and the result of our study (AUC of 0.82) was comparable to these markers. Multiparametric MRI scanning of prostate have been increasingly used for prostate cancer diagnosis in recent years [5]. Nevertheless, the availability of MRI for PCa diagnostic purposes is limited for less affluent or developing countries such as China due to the equipment requirements and high costs. Therefore, using machine learning techniques to maximize the predictive value of widely available clinical parameters would provide a cheaper and effective alternative to improve cancer prediction.

There are several noteworthy limitations of this study. First, the retrospective design resulted in many potential biases. For example, factors such as selection bias and variations in data collection methods will limit the validity of casual inference. Second, SHAP values illustrate relationships specific to a given model and dataset, and cannot be used to infer causality or underlying biological processes. Finally, both the training and test patient cohorts were from the same hospital at different time periods, and thus further multi-center external validation at other hospitals or regions is needed. Due to the limitation of database, the ability to predict clinically significant PCa was not reported in this study.

## Conclusion

Our machine learning model used routine serum biochemical markers to predict the risk of prostate cancer diagnosis with a high net benefit and avoid more unnecessary biopsies. The parameters included were objective and widely available, and the model can be used as a tool to optimize patient selection for further diagnosis, especially for those in underdeveloped or developing regions. P, K, CKMB, LDL-C, and Cre may be potential biochemical markers for predicting PCa, and risk thresholds for these markers were obtained using the SHAP method, which will be useful in diagnosis.

## Data Availability

The dataset analyzed during the current study is available[Web link to the dataset: https://www.ncmi.cn].

## Declarations

### Registry and the Registration No. Of the study/trial

N/A.

### Animal Studies

N/A.
